# A multi-site study on the impact of an advance care planning workshop on attitudes, beliefs and behavioural intentions over a 6-month period

**DOI:** 10.1186/s12909-021-02735-3

**Published:** 2021-05-25

**Authors:** C. C. Yu, E. J. Koh, J. A. Low, M. L. Ong, A. G. H. Sim, D. Y. Q. Hong, R. Chong, J. Low, R. Ng

**Affiliations:** 1 Geriatric Education and Research Institute, 2 Yishun Central 2, Singapore, 768024 Singapore; 2 Khoo Teck Puat Hospital, Singapore, Singapore; 3 Singapore General Hospital, Singapore, Singapore; 4 Tan Tock Seng Hospital, Singapore, Singapore; 5 Woodlands Health Campus, Singapore, Singapore

**Keywords:** Advance care planning, Evaluation, Healthcare professionals, Living matters, Training

## Abstract

**Background:**

This study evaluated the impact of the adapted version of the Respecting Choices® The Living Matters Advance Care Planning (ACP) facilitator training programme on trainees’ attitudes on facilitation 6 months post-training.

**Setting and participants:**

Two hundred and twenty-one healthcare professionals consisting of doctors, nurses, medical social workers from different training venues in Singapore participated in the first phase of the study (pre- and post) of which 107 participated in the second phase 6 months later (follow-up).

**Methods:**

Participants self-rated their attitudes, beliefs and behavioural intentions through surveys at three time points in an evaluation design that utilised repeated measures one-way ANOVA (pre-, post-, follow-up). Between-group differences were also examined using independent *t*-test.

**Results:**

At follow-up, mean scores increased significantly in understanding, confidence, and competence. Changes in effect sizes were large. Although trainees continued to think that ACP is emotionally draining for facilitators, more than before, facilitation experience was considered pleasant for themselves with the positive change significant and moderate in effect size. Those who had experience completing/initiating ACP significantly held more positive views than those who did not.

**Conclusions:**

The ACP facilitator training programme had lasting effects on enhancing the understanding, competence, and confidence of trainees. Importantly, findings showed that experience in actual facilitation within 6 months after training was important and giving trainees opportunities to facilitate is recommended.

**Supplementary Information:**

The online version contains supplementary material available at 10.1186/s12909-021-02735-3.

## Background

Advance Care Planning (ACP) is a process which involves individuals discussing and deciding on their future health care plans with healthcare professionals usually in the presence of their loved ones and/or future surrogate decision maker. The ACP and surrogate decision maker will help to voice the prior decisions of the individuals who have lost their mental capacity, and include discussions about personal values, goals of care, resuscitation and life support, palliative care options, proxy decision making, and advance directives [1]. Through ACP, such healthcare decisions will facilitate and ensure that the patient’s goals, values and preferences are honoured [2, 3].

ACP interventions that included the use of facilitated communication approaches are important [3, 4] and associated with positive outcomes in end-of-life care including a lower likelihood of dying in hospital and increased usage of palliative care, planned hospice and homecare services [4–6]. ACP has been known to reduce the likelihood of a patient receiving undesired treatments [6, 7] and improve patient and family satisfaction with care [6–8]. Despite the benefits, ACP discussions do not occur as frequently as they should [3, 9]. This is accounted for by the reluctance of patients and families to engage in such discussions, discomforts and concerns of physicians and other healthcare professionals over how patients will react, as well as beliefs or assumptions about the appropriateness of such discussions [10–15]. Whilst patient or organisational-related factors may subsequently affect attitudes of ACP facilitators, of particular concern here is whether such subjective attitudes and beliefs of healthcare professionals are adequately addressed by ACP training programmes in the first place.

Respecting Choices®, an internationally recognised ACP programme based in the United States, has been adapted in twelve countries including Australia, Canada and Germany [16]. This programme was adapted for use in Singapore in 2012 and is currently known as The Living Matters ACP programme. The facilitator training workshop of the programme aims to equip facilitators with knowledge, practical skills and process know how for ACP facilitation [17]. This study focused on examining the effects of the training on the facilitators through a multi-site study involving three acute care hospitals and a research institute based in Singapore.

Given that the ACP programme and training was relatively new in Asia at the time of this study in 2017, one of the key concerns then about the programme was whether a full-day workshop, focusing heavily on role-playing, was sufficient in building and sustaining a sense of confidence and competence in future ACP facilitators. For instance, it was unknown whether a short duration blended learning, flipped classroom approach would be effective compared to a traditional didactic classroom training approach. The primary research question addressed in this study was whether a full-day workshop with core content focusing on role playing, could effectively enhance understanding, competency, and confidence in facilitating ACP conversation 6 months post-training. The secondary aims were to (1) identify beliefs about ACP held by participants that may serve as a barrier to facilitation and (2) examine the effects of initiating and completing ACP on the ACP facilitator’s attitudes and beliefs.

## Methods

### Study design

Through opinions sought from ACP experts and trainers of the programme, a total of seven attitude domains were examined for this study. The focus was on self-perceived knowledge, skill competency, confidence, negative beliefs on ACP, positive beliefs on ACP, positive behavioural intentions, and beliefs on the benefits of ACP. Participants self-rated their attitudes, beliefs and behavioural intentions through surveys in an evaluation design. A repeated measures one-way ANOVA with Greenhouse-Geisser correction [18] was used to compare each measure across three time points; pre to post, post to follow-up, and pre to follow-up. Group differences from initiating or completing ACPs (yes vs. no) on these attitude domains were also examined using the independent *t*-test.

### Participants and procedures

Two hundred and twenty-three healthcare professionals who registered to attend ACP training between July 2017 to April 2018 from the three training sites in Singapore were recruited for the study. The pedagogy used was a flipped classroom approach. The programme consisted of two parts - pre-course online reading, followed by a one-day workshop which included didactic lectures, facilitation videos and role plays. The course objectives were as follows: (i) understanding the goals and scope of ACP, (ii) appreciating ethical considerations, and (iii) acquiring skills to conduct and facilitate an ACP discussion. Prior to attending the 1-day workshop, participants were asked to fill in a survey questionnaire, which was followed by two post-training surveys, one completed immediately after the workshop while the other 6 months later (refer to supplementary file for the pre-, post- and follow-up questionnaire developed for this study). The 6-month follow-up survey was administered via an online survey platform. Ethics approval was obtained from the National Healthcare Group Domain Specific Review Board (Ref no. 2017/00038). Written consent was obtained from all participants.

### Measures

All three surveys contained a series of questions that were developed for this study and participants were required to indicate the extent of agreement on a 4-point Likert-type scale, with anchors ranging from 1 (strongly disagree) to 4 (strongly agree). Measures were similar at all the three time points.

### Understanding of ACP

Perceived understanding of ACP was measured using a 5-item measure. Sample items included “I know what ACP is” and “I know the difference between ACP and other advance medical directives”. The internal reliability of this measure was good to excellent (α_pre_ = .82; α_post_ = .94; α_follow-up_ = .88).

### Skill competency

Perceived skill competency was measured using a 5-item measure. Sample items included “I am able to engage patients to introduce ACP to them” and “I am able to communicate about end-of-life care issues”. The internal reliability of this measure was good to excellent (α_pre_ = .87; α_post_ = .93; α_follow-up_ = .89).

### Overall confidence

Perceived overall confidence was measured using a 6-item measure. Sample items included “I feel confident responding to family members’ questions about ACP” and “I feel confident about conducting ACP discussions”. The internal reliability of this measure was good to excellent (α_pre_ = .93; α_post_ = .94; α_follow-up_ = .89).

### Positive behavioural intention

Positive behavioural intention was measured using a 3-item measure. Sample items included “I am enthusiastic about attending the ACP course” and “I am keen to facilitate ACP discussions with my patients”. The internal reliability of this measure was acceptable to good (α_pre_ = .83; α_post_ = .86; α_follow-up_ = .78).

### Beliefs on benefits of ACP

Beliefs on benefits of ACP was measured using a using a 3-item measure. Sample items included “I believe that doing ACP is helpful for my patients” and “I believe that patient’s family would find that ACP is useful”. The internal reliability of this measure was excellent (α_pre_ = .91; α_post_ = .96; α_follow-up_ = .91).

### Beliefs that ACP is emotionally draining for all

Beliefs that ACP is emotionally draining is a single item measure “Conducting an ACP discussion is emotionally draining for the ACP facilitator”.

### Beliefs that ACP is pleasant experience for self

Beliefs that ACP is a pleasant experience for self is a single item measure “Facilitating an ACP discussion is a pleasant experience for me”.

### Statistical package

Statistical analyses were conducted using Stata 14 software [19] and power analysis was conducted using G*Power 3 [20].

## Results

### Participant characteristics

Two hundred and twenty-one healthcare professionals from three ACP training venues in Singapore completed both questionnaires before and after the ACP training. Out of these study participants, 107 of them continued to participate in the online follow-up survey 6 months after the training. The proportional breakdown of sample characteristics in all questionnaires were similar (refer to Table 1). The mean age of the final sample of 107 participants (i.e., those who completed all three surveys) was 35.6 years (*SD* = 7.7) and majority of them were female (75.7%) and Chinese (74.8%). On average, participants had 5.3 years (*SD* = 5.1) of working experience and worked mostly in acute hospitals (61.7%). The bulk of the participants were nurses (45.8%) followed by doctors (24.3%) and medical social workers (23.4%).

**Table 1:** Demographic characteristics of trainees at the end of training (Pre and Post) vs. 6 months after training (Follow-up)

Characteristics	Pre and Post^a^		Follow-up	
	*N* = 221		*N* = 107	
Age	*X̅* = 34.6 years (*SD* = 7.5 years)		*X̅* = 35.6 years (*SD* = 7.7 years)	
Sex				
Female	166	(75.1%)	81	(75.7%)
Male	51	(23.1%)	24	(22.3%)
Unknown	4	(1.8%)	2	(2%)
Race				
Chinese	152	(68.8%)	79	(73.8%)
Filipino	21	(9.5%)	10	(9.3%)
Malay	19	(8.6%)	7	(6.5%)
Indian	17	(7.7%)	7	(6.5%)
Others	8	(3.6%)	4	(3.7%)
Unknown	4	(1.8%)	–	–
Marital Status				
Single	105	(47.5%)	53	(53.3)
Married	116	(52.5%)	54	(46.7)
Religion				
Christianity	68	(30.8%)	30	(28.0%)
Buddhism	42	(19.0%)	22	(20.6%)
Roman Catholicism	27	(12.2%)	14	(13.1%)
Islam	21	(9.5%)	8	(7.5%)
Hinduism	11	(5.0%)	4	(3.7%)
Taoism	3	(1.4%)	1	(.9%)
Others	5	(2.3%)	4	(3.7%)
No Religion	35	(15.8%)	20	(18.7%)
Unknown	9	(4.1%)	4	(3.7%)
Job Type				
Nurse	94	(42.5%)	49	(45.8%)
Doctor	71	(32.1%)	25	(24.3%)
Medical Social Worker	48	(21.7%)	26	(23.4%)
Unknown	8	(3.6%)	7	(6.5%)
Workplace				
Acute Hospital	137	(62.0%)	66	(61.7%)
Community Care	42	(19.0%)	23	(21.5%)
Community Hospital	13	(5.9%)	6	(5.6%)
Polyclinics	11	(5.0%)	5	(4.7%)
Others	5	(2.3%)	2	(1.9%)
Unknown	13	(5.9%)	5	(4.7%)
Total Years of Practice	*X̅* = 5.3 years (*SD* = 5.1 years)			*X̅* = 5.3 years (*SD* = 5.1 years)

Note: Community hospitals provide short-term therapy and treatment after patients have been discharged from an acute hospital. As the name implies, an acute hospital is one that sees medical cases of higher/increasing complexity

^a^ As the survey was administered once prior to the start (Pre) and another time immediately after (Post) the 1-day course, demographic profile was the same for both Pre and Post

### Attrition

Attrition was defined as participants who did not complete the entire set of evaluation in the two phases. The overall attrition rate for this study was therefore 51.5% (114/221).

### Training outcome

The means and standard deviations for each dependent variable at the 3 assessment points are shown in Table 2. For the following analysis, a repeated measures ANOVA was run for the group to determine if there were differences in perceptions, 6 months post-course.

**Table 2:** Means and standard deviations for outcome measures at pre-, post- training and 6-month follow-up evaluation

Variable	*n*	Mean	*SD*	Range
Understanding of ACP				
Pre	220	2.75	.42	1.6–4.0
Post	218	3.46	.48	1.0–4.0
Follow-up	105	3.23	.38	2.6–4.0
Perceived Skill Competency				
Pre	220	2.63	.50	1.0–4.0
Post	218	3.34	.48	1.0–4.0
Follow-up	105	3.14	.40	2.2–4.0
Overall Confidence				
Pre	218	2.55	.54	1.0–4.0
Post	216	3.24	.48	1.0–4.0
Follow-up	106	2.99	.50	2.0–4.0
Positive Behavioural Intention				
Pre	220	3.28	.43	1.3–4.0
Post	218	3.43	.47	2.3–4.0
Follow-up	105	3.25	.43	2.3–4.0
Beliefs on benefits of ACP				
Pre	221	3.33	.48	2.0–4.0
Post	217	3.52	.53	1.0–4.0
Follow-up	106	3.38	.45	3.0–4.0
Beliefs that ACP is emotionally draining for all				
Pre	218	2.69	.59	1.0–4.0
Post	217	2.69	.69	1.0–4.0
Follow-up	106	2.78	.65	1.0–4.0
Beliefs that ACP is Pleasant experience for self				
Pre	219	2.82	.64	1.0–4.0
Post	219	3.16	.65	1.0–4.0
Follow-up	106	2.97	.67	2.0–4.0

### Understanding of ACP

For the composite measure “Understanding of ACP”, the analysis ran on the 105 participants showed that the training programme elicited statistically significant differences in mean scores over time, *F*(2, 208) = 64.90, *p* < .001., partial _Ƞ_^2^ = .38, power = 1.00. Post hoc tests using Bonferroni correction revealed statistically significant results for all three time points. Although there was a small drop from post- to follow-up, overall, the knowledge gained from the training increased 6 months later as revealed in the scores for the pre- to follow-up evaluation and the effect size was large (see Table 3).

**Table 3:** Repeated measures ANOVA results by difference in scores at different time point

Measures	Mean Difference in score	*SE*	*t*	*p v*alue	95% CI of mean difference		*d*
					Lower Bound	Upper Bound	
Understanding of ACP							
Pre to Post	.62	.06	11.07	<.001*	.49	.76	1.27
Post to Follow-up	−.18	.06	−.3.22	.002†	.31	.58	−.40
Pre to Follow-up	.44	.05	7.86	<.001*	−.32	−.05	1.0
Perceived skill competency							
Pre to Post	.70	.05	13.95	<.001*	.58	.82	1.42
Post to Follow-up	−.14	.05	−2.74	.02†	−.26	−.02	−.31
Pre to Follow-up	.56	.05	11.21	<.001*	.44	.68	1.25
Overall confidence							
Pre to Post	.67	.06	11.78	<.001*	.53	.81	1.25
Post to Follow-up	−.17	.05	−2.95	.011†	−.31	−.03	−.34
Pre to Follow-up	.50	.06	8.83	<.001*	.37	.64	.94
Positive behavioural intention							
Pre to Post	.13	.05	2.55	.034†	.01	.24	.27
Post to Follow-up	−.18	.05	−3.60	.001*	−.29	−.06	−.39
Pre to Follow-up	−.05	.04	−1.05	.889	−.17	.07	−.12
Beliefs on benefits of ACP							
Pre to Post	.17	.05	3.07	.007†	.04	.31	.34
Post to Follow-up	−.16	.05	−2.90	.013†	−.30	−.03	−.33
Pre to Follow-up	.01	.04	.17	1.0	−.13	.14	.02
Beliefs that ACP is emotionally draining for all							
Pre to Post	.03	.07	.44	1.00	−.13	.19	.05
Post to Follow-up	.09	.07	1.33	.55	−.07	.25	.14
Pre to Follow-up	.12	.06	1.78	.23	−.04	.27	.18
Beliefs that ACP is pleasant experience for self							
Pre to Post	.42	.06	6.44	<.001*	.27	.58	.68
Post to Follow-up	−.19	.07	−2.86	.014†	−.35	−.03	−.29
Pre to Follow-up	.24	.06	3.58	.001†	.08	.40	.37

Note: contrasts subjected to Bonferroni correction and Cohen’s *d* indicate the standardised difference for each pair of means

* significant at the .001 level

† significant at the .01 level

### Perceived skills competency

For the composite measure “Perceived skills competency”, the analysis ran on the 105 participants showed that the training programme elicited statistically significant differences in mean scores over time, *F*(2, 208) = 109.25, *p* < .001., partial _Ƞ_^2^ = .51, power = 1.00. Post hoc tests using Bonferroni correction revealed statistically significant results for all three time points. Although there was a small drop from post- to follow-up, overall, the perception of one’s skill competency that results from the training increased 6 months later as revealed in the scores for the pre- to follow-up-evaluation and the effect size was large (see Table 3).

### Overall confidence

For the composite measure “Overall confidence”, the analysis ran on the 104 participants showed that the training programme elicited statistically significant differences in mean scores over time, *F*(2, 206) = 75.16, *p* < .001., partial _Ƞ_^2^ = .42, power = 1.00. Post hoc tests using Bonferroni correction revealed statistically significant results for all three time points. Although there was a small drop from post- to follow-up, overall, the perception of one’s skill competency that results from the training increased 6 months later as revealed in the scores for the pre- to follow-up-evaluation and the effect size was close to large (see Table 3).

### Positive behavioural intention

For the composite measure “Positive behavioural intention”, the analysis ran on the 104 participants showed that the training programme elicited statistically significant differences in mean scores over time, *F*(2, 206) = 6.85, *p* = .002, partial _Ƞ_^2^ = .06, power = .90. Post hoc tests using Bonferroni correction revealed significant time effects only from pre to post and post- to follow-up. Although there was a small increase from pre- to post, and a subsequent small decrease post-to follow-up, overall, positive behavioural intention was no different from baseline 6 months later (see Table 3).

### Beliefs on benefits of ACP

For the composite measure “Beliefs on benefits of ACP”, the analysis ran on the 105 participants showed that the training programme elicited statistically significant differences in mean scores over time *F*(2, 208) = 5.94, *p* = .016, partial _Ƞ_^2^ = .05, power = .85. Post hoc tests using Bonferroni correction revealed significant time effects only from pre to post and post- to follow-up. Although there was a small increase from pre- to post, and a subsequent small decrease post-to follow-up, overall, beliefs on benefits of ACP was no different from baseline 6 months later (see Table 3).

### Beliefs that ACP is emotionally draining for all

For the item measure “Beliefs that ACP is emotionally draining for all”, the analysis ran on the 103 participants showed that there were no difference in mean scores over time, *F*(2, 204) = 1.71, *p* = .194, partial _Ƞ_^2^ = .16, power = 1.00. The training did not have significant effects on perceptions that ACP is emotionally draining for everyone immediately after training or 6 months later (see Table 3).

### Beliefs that ACP is pleasant experience for self

For the item measure “Beliefs that ACP is pleasant experience for self”, the analysis ran on the 106 participants showed that the training programme elicited statistically significant differences in mean scores over time, *F*(2, 210) = 20.80, *p* < .001., partial _Ƞ_^2^ = .17, power = 1.00. Pairwise comparisons revealed statistically significant results for all three time points. Although there was a small drop from post- to follow-up, overall, beliefs that ACP is a pleasant experience for one’s own self increased 6 months later as revealed in the scores for the pre- to follow-up-evaluation and the effect size was moderate (see Table 3).

### Effects of initiating or completing ACP

To determine if attitudes differ 6 months after the training for those participants who had the opportunity to either initiate or complete ACP compared to those who do not, independent *t*-test was run to compare for between-group differences for the 7 attitudinal domains examined (refer to Tables 4 and 5). Results showed that those who either initiated or completed ACP had higher positive perceptions on the following domains: perceived skill competency, overall confidence, positive behavioural intention and beliefs that ACP is a pleasant experience for oneself. Additionally, those who initiated ACP had a better understanding of ACP. Overall, the effect sizes were between moderate to large.

**Table 4:** Results of *t*-tests and descriptive statistics of evaluation outcomes by ACP initiated within 6 months post training

Outcome	ACP initiated within 6 months post training						Mean Difference (95% CI for Mean Difference)			
	No			Yes						
	*M*	*SD*	*n*	*M*	*SD*	*n*		*t*	df	*d*
Understanding of ACP	3.11	.28	37	3.30	.41	66	−.27 (−.34, −.04)	−2.49*	101	−.51
Perceived Skill Competency	2.97	.36	38	3.24	.39	65	−.19 (−.43, −.12)	−3.52*	101	−.72
Overall Confidence	2.71	.45	38	3.17	.46	64	−.47 (−.65, −.28)	−4.99*	100	−1.02
Positive Behavioural Intention	3.14	.43	38	3.33	.42	65	−.19 (−.36, −.02)	−2.16*	101	−.44
Beliefs on benefits of ACP	3.33	.44	38	3.43	.46	65	−.09 (−.28, .09)	−1.00	101	−.20
Beliefs that ACP is emotionally draining for all	2.82	.61	38	2.75	.69	65	.06 (−.20, .33)	.46	101	.09
Beliefs that ACP is pleasant experience	2.66	.58	38	3.18	.64	66	−.52 (−.77, −.28)	−4.19*	102	−.85

* *p* value < .05 and significant at the .05 level

**Table 5:** Results of *t*-tests and descriptive statistics of evaluation outcomes by ACP completed within 6 months post training

Outcome	ACP completed within 6 months post training						Mean Difference (95% CI for Mean Difference)			
	No			Yes						
	*M*	*SD*	*n*	*M*	*SD*	*n*		*t*	df	*d*
Understanding of ACP	3.17	.33	66	3.29	.42	36	−.12 (−.27, .03)	−1.53	100	−.32
Perceived Skill Competency	3.04	.37	67	3.27	.38	35	−.23 (−.39, −.08)	−2.97*	100	−.62
Overall Confidence	2.86	.49	66	3.21	.44	35	−.34 (−.54, −.15)	−3.48*	99	−.73
Positive Behavioural Intention	3.17	.41	66	3.38	.41	36	−.21 (−.38, −.04)	−2.49*	100	−.52
Beliefs on benefits of ACP	3.33	.45	67	3.49	.45	35	−.16 (−.34, .03)	−1.69	100	−.35
Beliefs that ACP is emotionally draining for all	2.83	.62	66	2.69	.67	36	.14 (−.12, .40)	1.05	100	.22
Beliefs that ACP is pleasant experience	2.78	.62	67	3.31	.58	36	−.53 (−.78, −.28)	−4.22*	101	−.87

* *p* value < .05 and significant at the .05 level

Given the presence of small effect sizes for the remaining variables, the modest sample size played a role in limiting the significance of the statistical comparison. For instance, *t*-test results for the attitude domain “beliefs that ACP is emotionally draining” indicated that individuals who did not complete any ACP (*M* = 2.83, *SD* = .63, *n* = 67) held higher agreement scores on emotionally draining beliefs compared to those who completed ACP (*M* = 2.69, *SD* = .67, *n* = 37). The difference of .14, 95% CI (−.12 to 40) was small and not significant, *t*(105) = 1.05, *p* > .05. A post-hoc power analysis revealed that the obtained power for this analysis was at .18, much lower than the recommended level of .80 [21]. With a larger sample size, odds for statistical significance will increase and those who initiated and completed ACP are expected to hold positive beliefs on benefits of ACP and less likely to think that ACP is emotionally draining although effect of the difference is practically small.

## Discussion

This study evaluated the effectiveness of The Living Matters ACP facilitator training workshop in Singapore. The training, modelled after the flipped classroom approach, which consisted of pre-course reading materials and a full-day workshop with its core content focusing on role playing, was shown to enhance the understanding, competency, and confidence of the trainees. This was demonstrated through statistically significant increase in self-reported mean scores for these measures, across time points (post-course and follow-up 6 months later) with notably large effect sizes. Concerns about the adequacy of the duration, content, or pedagogical approach of this training programme in building up the proficiency of the trainees were therefore addressed in this study.

Although the training did not appear to bring large changes to behavioural intentions such as keenness to facilitate ACP or alter existing beliefs on the benefits of ACP, this was possibly accounted for by ceiling effects whereby participants held favourable views even before the training. As such, it cannot be ruled out that self-selection bias could have contributed to this and those who joined this particular programme and participated throughout this study held very positive views about ACP. The study team noted through a post hoc study review that views may possibly differ depending on whether the participants attended the course voluntarily. As this information was not formally collected from the participants through the surveys, this remained a postulation. Future evaluation research in this domain may need to consider if participants signed up voluntarily or were tasked to attend by their organisations.

As with previous studies [22], there was the prevailing sense that conducting ACP discussions was generally a discomforting experience for facilitators. This was borne out by findings from this study that beliefs about ACP as being emotionally draining did not change 6 months after the course. However, rather than issues with training, it could be argued that organisational or structural factors contributed to such views. For instance, the lack of a facilitator-mentor and ‘handholding’ system by the organisation where the newly trained facilitator is situated, the absence of peer support groups and the limited protected time provided to conduct ACP outside of the primary work of facilitators could potentially contribute to such ‘emotionally draining’ experiences. Being part of an institution or department that supports and encourages ACP could therefore be a key to ACP facilitators initiating and completing ACP conversations with their patients. Future training programmes should not end with the workshops but should perhaps extend beyond the classroom into the real clinical world. A post-course support module (e.g., assignment of experienced mentors at work, online videos to deal with common barriers, case-based discussions, etc.) could be further developed in alignment with this.

An important contribution from this study was to show that the opportunity to initiate and/or complete ACP made a difference in developing positive subjective experiences despite the generally held belief that ACP discussions may be distressing. Having such an opportunity led to trainees reporting that facilitation was more pleasant for them at the 6-month follow-up. Likewise, subjective experiences were also more positive in the domains of understanding, confidence, and competence. In all, effect sizes were observed to range from moderate to large. To actualise the benefits of an ACP training programme, having a component that allows some practice experience in the healthcare and community setting may be beneficial. Perhaps, a more experiential teaching methodology using an ontological approach could also be implemented to enhance the training pedagogy. The selection process of participants and future ACP facilitators should also be carried out in a considered way where those who will initiate ACP conversations in the upcoming 6 months should be given priority as they are the most likely candidates to benefit from the workshop compared to those who will do so at a much later period.

This study has its limitations with the presence of several threats to internal validity. The first threat relates to mortality. The attrition rate in the follow-up phase was high and hence it was difficult to determine whether those who chose to continue with the study were different from those lost. This threat was difficult to address as attrition was expected for this form of data collection method. Analyses, however, did show that sample characteristics in all the three phases were largely similar, reducing the odds that views may differ due to differences in demographic profile of the participants (e.g., education, job scope or workplace). As part of study protocol, the study team made up to three attempts to contact participants 6 months post training to continue with the survey. Whilst following up on these participants would be important, the study protocol did not allow the study team to re-contact them should they choose not to reply following the three attempts. The second threat pertains to practice effects. Although participants were aware of the purpose of the study, with no implication on their performance assessment, practice effects could have occurred as a result of answering the same set of questions over three time points. Participants may have assumed that it is ideal to carry-over and improve on their responses over time. The third threat relates to maturation effects and the positive findings at the follow-up phase could in part be attributed to this. Unrelated to the training provided 6 months ago, growth and change from gaining experience at the workplace may have impacted understanding, confidence, and competence levels. This threat would be difficult to address and expected for real world research [23] given that this study was conducted in the context of an applied setting where controlling environmental influences would neither be practical nor feasible. Likewise, for the same reasons, it would be difficult to address history effects whereby possible events or circumstances in the environment affected the responses of some or all of the participants at the 6 months follow-up phase. Finally, this study could also have included validated tools that measures perceived self-efficacy in the education setting. Including such measures would allow the comparison of findings under different cultural or institutional settings.

## Conclusion

In summary, the Living Matters ACP facilitator training programme was effective in increasing the understanding, competency, and confidence of the trainees with effects that were moderate to strong and potentially long lasting. This study is important as ACP facilitator training is relatively new in Asia and assuages the concerns over the effectiveness of a one-day course that utilises the flipped classroom approach. Findings from this study suggest that to enhance the effectiveness accrued from training, an ACP training programme can be enhanced by having a component that allows some practice opportunity in the healthcare and community setting within 6 months of training. Future studies on ACP training could consider examining the effects of structured post-training support such as handholding or mentoring by an experienced facilitator. Additional training support which is more sustained, with resources such as videos, hotlines, printed materials, could be studied to see if they are able to ease some of the discomforts and challenges associated with ACP facilitation.

## Supplementary Information

**Additional file 1.** Pre-course questionnaire.

**Additional file 2.** Post-course questionnaire.

**Additional file 3.** Follow-up questionnaire.

## Data Availability

The datasets used and/or analysed during the current study are available from the corresponding author on reasonable request*.*
