# Correction to: A multi-site study on the impact of an advance care planning workshop on attitudes, beliefs and behavioural intentions over a 6-month period

**DOI:** 10.1186/s12909-021-02816-3

**Published:** 2021-07-08

**Authors:** C. C. Yu, E. J. Koh, J. A. Low, M. L. Ong, A. G. H. Sim, D. Y. Q. Hong, R. Chong, J. Low, R. Ng

**Affiliations:** 1 Geriatric Education and Research Institute, 2 Yishun Central 2, Singapore, 768024 Singapore; 2 Khoo Teck Puat Hospital, Singapore, Singapore; 3 Singapore General Hospital, Singapore, Singapore; 4 Tan Tock Seng Hospital, Singapore, Singapore; 5 Woodlands Health Campus, Singapore, Singapore

**Correction to: BMC Med Educ 21, 298 (2021)**

**https://doi.org/10.1186/s12909-021-02735-3**

Following publication of the original article [1], we have been informed that the author J. Low had the affiliation incorrectly assigned.

The author group has been updated above and the original article [1] has been corrected.
