# Supplementary material for: A multi-site study on the impact of an advance care planning workshop on attitudes, beliefs and behavioural intentions over a 6-month period
Source: BMC Med Educ. 2021 May 25;21:298. doi: 10.1186/s12909-021-02735-3 (PMC8146668; doi:10.1186/s12909-021-02735-3)
Supplement: Supplementary file 1 — Additional file 1. Pre-course questionnaire. [file 12909_2021_2735_MOESM1_ESM.pdf]

A Mixed Methods Evaluation of the Living Matters Advance Care Planning (ACP) training course

Questionnaire for Participants

Demographics

Please **fill in** your information below:

Year of Birth: _____________ **Gender:** M / F

Marital Status: _____________ **Race:** ____________________ **Religion:** _________________

Designation/Occupation: ______________ **Primary workplace:** __________________________________

Years of practice in current workplace: ______ Year(s) ______ Month(s)

Knowledge

For each of the statements below, **circle** the correct answer (True/False):

1	The best time to discuss ACP is when patients are seriously ill.	True/False
2	ACP is a legal document.	True/False
3	The nominated healthcare spokesperson must be a family member.	True/False
4	ACP is a one-time event.	True/False
5	The doctor can choose not to follow the decisions made in the ACP document.	True/False
6	For an effective ACP discussion, one of the criteria to ask the patient is to identify a trusted individual as his or her healthcare spokesperson.	True/False
7	An ACP document is valid only if completed by an adult who has decision making capacity.	True/False
8	An ACP document can be only activated in the event that the person has lost decision making capacity.	True/False
9	An ACP document is only referred to when the person has lost decision making capacity.	True/False
10	An individual's beliefs significantly affects the preferences for treatment discussed in ACP.	True/False
11	ACP is always a part of routine health care.	True/False
12	ACP can be completed without a nominated healthcare spokesperson.	True/False
13	Understanding the prognosis, options for care and background of the ACP participant is vital for a successful ACP discussion.	True/False
14	The nominated healthcare spokesperson needs to be someone who is able to speak on patient's behalf under stressful situations.	True/False
15	A person with dementia is deemed to have no mental capacity to make an ACP.	True/False
16	Ability to reason and to weigh treatment options are part of the requirements for a person to possess decision-making capacity.	True/False
17	A patient is assumed to have decision making capacity unless proven otherwise.	True/False
18	One can presume that decision-making capacity is absent by looking at a person's age, diagnosis, behaviour or appearance.	True/False
19	ACP is preferably done in the inpatient setting than in the outpatient clinic.	True/False
20	The process of ACP involves individualizing the discussion to the individual's state of health.	True/False

Confidence in Knowledge/Perceived Knowledge

For each of the statements below, **circle** the number that best describes your opinion:

	Strongly Agree	Agree	Disagree	Strongly Disagree
1. I know what ACP is ……………………………………………………………..	1	2	3	4
2. I know who to introduce ACP to. ………………………………………..	1	2	3	4
3. I know when to conduct an ACP discussion…………………………	1	2	3	4
4. I know the difference between ACP and other advance medical directives. ……………………………………………………………..	1	2	3	4
5. I understand the ACP framework and the different types of ACP.……………………………………………………………………………….	1	2	3	4

Skills

For each of the statements below, **circle** the number that best describes your opinion:

	Strongly Agree	Agree	Disagree	Strongly Disagree
1. I am able to engage patients to introduce ACP to them………	1	2	3	4
2. I am able to advise patients if they are keen to make an ACP. ……………………………………………………………………………….	1	2	3	4
3. I am able to share the key issues in ACP within the localised context and framework used in Singapore………………………….	1	2	3	4
4. I am able to communicate about end-of-life care issues……..	1	2	3	4
5. I am able to identify and use appropriate resources to provide information about ACP.…………………………………………	1	2	3	4

Attitudes

For each of the statements below, **circle** the number that best describes your opinion:

	Strongly Agree	Agree	Disagree	Strongly Disagree
1. I am enthusiastic about attending the ACP course……………..	1	2	3	4
2. I am keen to facilitate ACP discussions with my patients……	1	2	3	4
3. Facilitating an ACP discussion is a pleasant experience for me………………………………………………………………………………	1	2	3	4
4. I believe that doing ACP is helpful for my patients……………..	1	2	3	4
5. I believe that patients would find that ACP is useful………….	1	2	3	4
6. I believe that patient's family would find that ACP is useful..	1	2	3	4
7. I think I will recommend my colleagues to attend the ACP course as well…………………………………………………………………….	1	2	3	4
8. Patients with decision-making capacity should have a right to refuse life sustaining treatment even if that decision may lead to adverse outcomes. ………………………………………………	1	2	3	4
9. Healthcare providers should respect a patient's wishes even if they conflict with the family's wishes…………………….	1	2	3	4
10. Healthcare providers should be actively involved in facilitating patients complete ACP……………………………………	1	2	3	4
11. Healthcare providers should disregard a patient's ACP if they think the treatment is in the patient's best interests….	1	2	3	4
12. Healthcare providers should help inform patients about their condition and treatment alternatives when preparing an ACP…………………………………………………………………………….	1	2	3	4
13. Healthcare providers usually know the wishes of their patients regarding end-of-life care without having formal documentation…………………………………………………………………….	1	2	3	4
14. The information in an ACP is usually sufficient to guide treatment………………………………………………………………………….	1	2	3	4
15. Most of the time family members know the patient's preference regarding end-of –life care…………………………….	1	2	3	4
16. Conducting an ACP discussion is emotionally draining for the ACP facilitator……………………………………………………………….	1	2	3	4
17. I feel that a patient's ACP decisions will be honoured by healthcare professionals…………………………………………………….	1	2	3	4

Confidence

For each of the statements below, **circle** the number that best describes your confidence

	Strongly Agree	Agree	Disagree	Strongly Disagree
1. I feel confident about introducing the concept of ACP……….	1	2	3	4
2. I feel confident about conducting ACP discussions………………	1	2	3	4
3. I feel confident discussing a patient's illness and treatment options in relation to ACP…………………………………………………	1	2	3	4
4. I feel confident having the skills to discuss ACP………………….	1	2	3	4
5. I feel confident answering patients' questions about ACP…..	1	2	3	4
6. I feel confident responding to family members' questions about ACP……………………………………………………..…………………….	1	2	3	4
