# Supplementary material for: A multi-site study on the impact of an advance care planning workshop on attitudes, beliefs and behavioural intentions over a 6-month period
Source: BMC Med Educ. 2021 May 25;21:298. doi: 10.1186/s12909-021-02735-3 (PMC8146668; doi:10.1186/s12909-021-02735-3)
Supplement: Supplementary file 2 — Additional file 2. Post-course questionnaire. [file 12909_2021_2735_MOESM2_ESM.pdf]

A Mixed Methods Evaluation of the Living Matters Advance Care Planning (ACP) training course

Questionnaire for Participants

Demographics

Please **fill in** your information below:

Year of Birth: ______________ **Gender:** M / F

Marital Status: _____________ **Race:** _____________________ **Religion:** __________________

Designation/Occupation: _______________ **Primary workplace:** ____________________________________

Years of practice in current workplace: ______ Year(s) ______ Month(s)

Knowledge

For each of the statements below, **circle** the correct answer (True/False):

1	The best time to discuss ACP is when patients are seriously ill.	True/False
2	ACP is a legal document.	True/False
3	The nominated healthcare spokesperson must be a family member.	True/False
4	ACP is a one-time event.	True/False
5	The doctor can choose not to follow the decisions made in the ACP document.	True/False
6	For an effective ACP discussion, one of the criteria to ask the patient is to identify a trusted individual as his or her healthcare spokesperson.	True/False
7	An ACP document is valid only if completed by an adult who has decision making capacity.	True/False
8	An ACP document can be only activated in the event that the person has lost decision making capacity.	True/False
9	An ACP document is only referred to when the person has lost decision making capacity.	True/False
10	An individual’s beliefs significantly affects the preferences for treatment discussed in ACP.	True/False
11	ACP is always a part of routine health care.	True/False
12	ACP can be completed without a nominated healthcare spokesperson.	True/False
13	Understanding the prognosis, options for care and background of the ACP participant is vital for a successful ACP discussion.	True/False
14	The nominated healthcare spokesperson needs to be someone who is able to speak on patient’s behalf under stressful situations.	True/False
15	A person with dementia is deemed to have no mental capacity to make an ACP.	True/False
16	Ability to reason and to weigh treatment options are part of the requirements for a person to possess decision-making capacity.	True/False
17	A patient is assumed to have decision making capacity unless proven otherwise.	True/False
18	One can presume that decision-making capacity is absent by looking at a person’s age, diagnosis, behaviour or appearance.	True/False
19	ACP is preferably done in the inpatient setting than in the outpatient clinic.	True/False
20	The process of ACP involves individualizing the discussion to the individual’s state of health.	True/False

Confidence in Knowledge/Perceived Knowledge

For each of the statements below, **circle** the number that best describes your opinion:

	Strongly Agree	Agree	Disagree	Strongly Disagree
1. I know what ACP is …………………………………………………………….	1	2	3	4
2. I know who to introduce ACP to. ……………………………………….	1	2	3	4
3. I know when to conduct an ACP discussion……………………….	1	2	3	4
4. I know the difference between ACP and other advance medical directives. …………………………………………………………….	1	2	3	4
5. I understand the ACP framework and the different types of ACP…………………………………………………………………………….	1	2	3	4

Skills

For each of the statements below, **circle** the number that best describes your opinion:

	Strongly Agree	Agree	Disagree	Strongly Disagree
1. I am able to engage patients to introduce ACP to them………	1	2	3	4
2. I am able to advise patients if they are keen to make an ACP. …………………………………………………………………………….	1	2	3	4
3. I am able to share the key issues in ACP within the localised context and framework used in Singapore……………………….	1	2	3	4
4. I am able to communicate about end-of-life care issues…….	1	2	3	4
5. I am able to identify and use appropriate resources to provide information about ACP………………………………………….	1	2	3	4

Attitudes

For each of the statements below, **circle** the number that best describes your opinion:

	Strongly Agree	Agree	Disagree	Strongly Disagree
1. I am enthusiastic about attending the ACP course................	1	2	3	4
2. I am keen to facilitate ACP discussions with my patients......	1	2	3	4
3. Facilitating an ACP discussion is a pleasant experience for me..	1	2	3	4
4. I believe that doing ACP is helpful for my patients................	1	2	3	4
5. I believe that patients would find that ACP is useful.............	1	2	3	4
6. I believe that patient's family would find that ACP is useful..	1	2	3	4
7. I think I will recommend my colleagues to attend the ACP course as well...	1	2	3	4
8. Patients with decision-making capacity should have a right to refuse life sustaining treatment even if that decision may lead to adverse outcomes.	1	2	3	4
9. Healthcare providers should respect a patient's wishes even if they conflict with the family's wishes.........................	1	2	3	4
10. Healthcare providers should be actively involved in facilitating patients complete ACP...	1	2	3	4
11. Healthcare providers should disregard a patient's ACP if they think the treatment is in the patient's best interests....	1	2	3	4
12. Healthcare providers should help inform patients about their condition and treatment alternatives when preparing an ACP...	1	2	3	4
13. Healthcare providers usually know the wishes of their patients regarding end-of-life care without having formal documentation...	1	2	3	4
14. The information in an ACP is usually sufficient to guide treatment..	1	2	3	4
15. Most of the time family members know the patient's preference regarding end-of –life care..................................	1	2	3	4
16. Conducting an ACP discussion is emotionally draining for the ACP facilitator...	1	2	3	4
17. I feel that a patient's ACP decisions will be honoured by healthcare professionals..	1	2	3	4

Confidence

For each of the statements below, **circle** the number that best describes your confidence

	Strongly Agree	Agree	Disagree	Strongly Disagree
1. I feel confident about introducing the concept of ACP……….	1	2	3	4
2. I feel confident about conducting ACP discussions……………..	1	2	3	4
3. I feel confident discussing a patient's illness and treatment options in relation to ACP………………………………………………	1	2	3	4
4. I feel confident having the skills to discuss ACP…………………	1	2	3	4
5. I feel confident answering patients' questions about ACP…..	1	2	3	4
6. I feel confident responding to family members' questions about ACP………………………………………………………………	1	2	3	4

Satisfaction

For each of the statements below, **circle** the number that best describes your satisfaction with the workshop?

	Strongly Agree	Agree	Disagree	Strongly Disagree
1. The training was adequate for me to introduce ACP to my patients and their family. ………………………………………………	1	2	3	4
2. The topics covered were relevant for my work………………..	1	2	3	4
3. The role-plays were helpful in developing my confidence to conduct ACP discussions……………………………………………….	1	2	3	4
4. The readings were at a right level for the difficulty of the course………………………………………………………………………	1	2	3	4
5. The materials were presented in a way that helps me learn.	1	2	3	4
6. The presentations were interesting and helpful. …………….	1	2	3	4
7. The pace of the workshop was appropriate. …………………..	1	2	3	4
8. Attending the course has increased my confidence level in conducting ACP discussions…………………………………………….	1	2	3	4
9. The training programme was well organized……………………	1	2	3	4
10. Opportunities for participation were appropriate……………	1	2	3	4
11. I felt able to contribute to group discussions. …………………	1	2	3	4
12. The resource file was easy to understand………………………	1	2	3	4
13. The resource file stimulated discussion …………………………	1	2	3	4

Components of the course

1. Please **rate** the quality of the following:

	Poor	Fair	Good	Very Good	Excellent
Overall content of course					
E-learning materials					
Pre-reading materials					
PowerPoint slides					
Videos					
Lectures					
Resource file					
Role play					

Overall Experience

1. What did you **enjoy most** about the overall experience of the training programme?

____

____

____

2. What is the **most significant thing** that you have **learnt** after the workshop?

____

____

____

3. How has the workshop **changed your thinking** regarding ACP facilitation?

____

____

____
