# Supplementary material for: A multi-site study on the impact of an advance care planning workshop on attitudes, beliefs and behavioural intentions over a 6-month period
Source: BMC Med Educ. 2021 May 25;21:298. doi: 10.1186/s12909-021-02735-3 (PMC8146668; doi:10.1186/s12909-021-02735-3)
Supplement: Supplementary file 3 — Additional file 3. Follow-up questionnaire. [file 12909_2021_2735_MOESM3_ESM.pdf]

# A Mixed Methods Evaluation of the Living Matters Advance Care Planning (ACP) training course

Questionnaire for Participants

## Demographics

Please **fill in** your information below:

**Year of Birth:** _____________ **Gender:** M / F

**Marital Status:** _____________ **Race:** ____________________ **Religion:** _________________

**Designation/Occupation:** _______________ **Primary workplace:** ____________________________________

**Years of practice in current workplace:** ______ Year(s) ______ Month(s)

## Knowledge

For each of the statements below, **circle** the correct answer (True/False):

| | | |
|---|---|---|
| 1 | The best time to discuss ACP is when patients are seriously ill. | True/False |
| 2 | ACP is a legal document. | True/False |
| 3 | The nominated healthcare spokesperson must be a family member. | True/False |
| 4 | ACP is a one-time event. | True/False |
| 5 | The doctor can choose not to follow the decisions made in the ACP document. | True/False |
| 6 | For an effective ACP discussion, one of the criteria to ask the patient is to identify a trusted individual as his or her healthcare spokesperson. | True/False |
| 7 | An ACP document is valid only if completed by an adult who has decision making capacity. | True/False |
| 8 | An ACP document can be only activated in the event that the person has lost decision making capacity. | True/False |
| 9 | An ACP document is only referred to when the person has lost decision making capacity. | True/False |
| 10 | An individual's beliefs significantly affects the preferences for treatment discussed in ACP. | True/False |
| 11 | ACP is always a part of routine health care. | True/False |
| 12 | ACP can be completed without a nominated healthcare spokesperson. | True/False |
| 13 | Understanding the prognosis, options for care and background of the ACP participant is vital for a successful ACP discussion. | True/False |
| 14 | The nominated healthcare spokesperson needs to be someone who is able to speak on patient's behalf under stressful situations. | True/False |
| 15 | A person with dementia is deemed to have no mental capacity to make an ACP. | True/False |
| 16 | Ability to reason and to weigh treatment options are part of the requirements for a person to possess decision-making capacity. | True/False |
| 17 | A patient is assumed to have decision making capacity unless proven otherwise. | True/False |
| 18 | One can presume that decision-making capacity is absent by looking at a person's age, diagnosis, behaviour or appearance. | True/False |
| 19 | ACP is preferably done in the inpatient setting than in the outpatient clinic. | True/False |
| 20 | The process of ACP involves individualizing the discussion to the individual's state of health. | True/False |

## ***Confidence in Knowledge/Perceived Knowledge***

For each of the statements below, **circle** the number that best describes your opinion:

| | Strongly Agree | Agree | Disagree | Strongly Disagree |
|---|---|---|---|---|
| 1. I know what ACP is ………………………………………………………….. | 1 | 2 | 3 | 4 |
| 2. I know who to introduce ACP to. ………………………………………. | 1 | 2 | 3 | 4 |
| 3. I know when to conduct an ACP discussion………………………. | 1 | 2 | 3 | 4 |
| 4. I know the difference between ACP and other advance medical directives. ……………………………………………………………. | 1 | 2 | 3 | 4 |
| 5. I understand the ACP framework and the different types of ACP……………………………………………………………………………. | 1 | 2 | 3 | 4 |

## ***Skills***

For each of the statements below, **circle** the number that best describes your opinion:

| | Strongly Agree | Agree | Disagree | Strongly Disagree |
|---|---|---|---|---|
| 1. I am able to engage patients to introduce ACP to them……… | 1 | 2 | 3 | 4 |
| 2. I am able to advise patients if they are keen to make an ACP. …………………………………………………………………………….. | 1 | 2 | 3 | 4 |
| 3. I am able to share the key issues in ACP within the localised context and framework used in Singapore……………………….. | 1 | 2 | 3 | 4 |
| 4. I am able to communicate about end-of-life care issues…….. | 1 | 2 | 3 | 4 |
| 5. I am able to identify and use appropriate resources to provide information about ACP………………………………………. | 1 | 2 | 3 | 4 |

## ***Attitudes***

For each of the statements below, **circle** the number that best describes your opinion:

| | Strongly Agree | Agree | Disagree | Strongly Disagree |
|---|---|---|---|---|
| 1. I am enthusiastic about attending the ACP course…………….. | 1 | 2 | 3 | 4 |
| 2. I am keen to facilitate ACP discussions with my patients…… | 1 | 2 | 3 | 4 |
| 3. Facilitating an ACP discussion is a pleasant experience for me…………………………………………………………………………… | 1 | 2 | 3 | 4 |
| 4. I believe that doing ACP is helpful for my patients……………. | 1 | 2 | 3 | 4 |
| 5. I believe that patients would find that ACP is useful…………. | 1 | 2 | 3 | 4 |
| 6. I believe that patient's family would find that ACP is useful.. | 1 | 2 | 3 | 4 |
| 7. I think I will recommend my colleagues to attend the ACP course as well……………………………………………………………. | 1 | 2 | 3 | 4 |
| 8. Patients with decision-making capacity should have a right to refuse life sustaining treatment even if that decision may lead to adverse outcomes. ……………………………………………… | 1 | 2 | 3 | 4 |
| 9. Healthcare providers should respect a patient's wishes even if they conflict with the family's wishes……………………. | 1 | 2 | 3 | 4 |
| 10. Healthcare providers should be actively involved in facilitating patients complete ACP…………………………………. | 1 | 2 | 3 | 4 |
| 11. Healthcare providers should disregard a patient's ACP if they think the treatment is in the patient's best interests…. | 1 | 2 | 3 | 4 |
| 12. Healthcare providers should help inform patients about their condition and treatment alternatives when preparing an ACP…………………………………………………………………………. | 1 | 2 | 3 | 4 |
| 13. Healthcare providers usually know the wishes of their patients regarding end-of-life care without having formal documentation…………………………………………………………… | 1 | 2 | 3 | 4 |
| 14. The information in an ACP is usually sufficient to guide treatment………………………………………………………………………. | 1 | 2 | 3 | 4 |
| 15. Most of the time family members know the patient's preference regarding end-of –life care……………………………. | 1 | 2 | 3 | 4 |
| 16. Conducting an ACP discussion is emotionally draining for the ACP facilitator………………………………………………………… | 1 | 2 | 3 | 4 |
| 17. I feel that a patient's ACP decisions will be honoured by healthcare professionals…………………………………………………. | 1 | 2 | 3 | 4 |

## ***Confidence***

For each of the statements below, **circle** the number that best describes your confidence

| | Strongly Agree | Agree | Disagree | Strongly Disagree |
|---|---|---|---|---|
| 1. I feel confident about introducing the concept of ACP………. | 1 | 2 | 3 | 4 |
| 2. I feel confident about conducting ACP discussions……………… | 1 | 2 | 3 | 4 |
| 3. I feel confident discussing a patient's illness and treatment options in relation to ACP………………………………………………… | 1 | 2 | 3 | 4 |
| 4. I feel confident having the skills to discuss ACP………………… | 1 | 2 | 3 | 4 |
| 5. I feel confident answering patients' questions about ACP….. | 1 | 2 | 3 | 4 |
| 6. I feel confident responding to family members' questions about ACP………………………………………………..……………………. | 1 | 2 | 3 | 4 |

## ***Practice***

In the past 6 months:

1. Have you **initiated** any ACP discussions? (Yes / No)

   If yes, please state the number: _____

2. Have you **completed** any ACP discussions? (Yes / No)

   If yes, please state the number: ____

3. Have you **observed treatment** being provided that was not reflective of a patient's wishes? ( Yes/ No)
